# Optimization of layering technique and secondary structure analysis during the formulation of nanoparticles containing lysozyme by quality by design approach

**DOI:** 10.1371/journal.pone.0260603

**Published:** 2021-12-09

**Authors:** Katalin Kristó, Reihaneh Manteghi, Yousif H-E. Y. Ibrahim, Ditta Ungor, Edit Csapó, Dániel Berkesi, Zoltán Kónya, Ildikó Csóka

**Affiliations:** 1 Institute of Pharmaceutical Technology and Regulatory Affairs, University of Szeged, Szeged, Hungary; 2 Department of Physical Chemistry and Materials Science, MTA-SZTE Lendület “Momentum” Noble Metal Nanostructures Research Group, Interdisciplinary Excellence Center, University of Szeged, Szeged, Hungary; 3 Department of Medical Chemistry, MTA-SZTE Biomimetic Systems Research Group, University of Szeged, Szeged, Hungary; 4 Department of Applied and Environmental Chemistry, University of Szeged, Szeged, Hungary; Hamadan University of Medical Sciences, ISLAMIC REPUBLIC OF IRAN

## Abstract

In our study, core-shell nanoparticles containing lysozyme were formulated with precipitation and layering self-assembly. Factorial design (DoE) was applied by setting the process parameters during the preparation with Quality by Design (QbD) approach. The factors were the concentration of lysozyme and sodium alginate, and pH. Our aim was to understand the effect of process parameters through the determination of mathematical equations, based on which the optimization parameters can be predicted under different process parameters. The optimization parameters were encapsulation efficiency, particle size, enzyme activity and the amount of α-helix structure. The nanoparticles were analysed with transmission electron microscopy (TEM), Fourier-transform infrared spectroscopy (FTIR) and circular dichroism (CD) spectroscopy. Based on our results, we found that pH was the most important factor and pH 10 was recommended during the formulation. Enzyme activity and α-helix content correlated with each other very well, and particle size and encapsulation efficiency also showed very good correlation with each other. The results of the α-helix content of FTIR and CD measurements were very similar for the precipitated lysozyme due to the solid state of lysozyme. The mixing time had the best influence on the encapsulation efficiency and the particle size, which leads to the conclusion that a mixing time of 1 h is recommended. The novelty in our study is the presentation of a mathematical model with which the secondary structure of the protein and other optimization parameters can be controlled in the future during development of nanoparticle based on the process parameters.

## Introduction

Recently, the use of proteins in the biomedical field has become more extensive, and they have been investigated intensively as potential biopharmaceutical drugs [1]. They have well-known high specificity, complexity and low toxicity compared to small drug entities, but at the same time a number of barriers need to be overcome for the development of stably absorbable delivery systems [2].

Lysozyme (LYS) is a harmless natural antimicrobial enzyme protein that can be derived from plants, animals and microorganisms as a single chain polypeptide. It has a globular shape constructed of 129 amino acids with an approximate molecular weight of 14 kDa, with a quite alkaline nature of 11 isoelectric points, its main physiological role is to perform the host`s natural immune defence effect [3, 4]. Hen-egg white is a common source in LYS separation studies, which is mainly performed by the precipitation method of egg-white proteins upon the addition of salts, solvents or the reduction of ionic strength [5]. The diversity of the source renders LYS more affordable and cost-effective protein for the investigations [6]. The antimicrobial activity of lysozyme is attributed to the destruction and lysation of the cell wall of Gram-positive bacteria and some fungi [7]. Moreover, *in vitro* studies prove the activity of LYS against many Gram-negative bacteria including Pseudomonas aeruginosa [8].

Pharmaceutically, nanotechnology has been performed to improve drug delivery performance, basically by improving bioavailability through the administration of the drug entity in nanoscale particles (NPs) or molecules (in the range of 1-1000nm) which can overcome the biological barriers, targeting the absorption site, enhancing stability and solubility by increasing the surface area [9]. Therefore, various nanotechnology-based drug formulations have been introduced to the market for treating and controlling numerous diseases, such as cancer, central nervous system diseases and infections [10]. Moreover, the delivery of the drug in the form of NPs protects the natural products, such as proteins, from degrading enzymes, as well as controls the release of the incorporated bioactive molecules [11].

Nanocarrier systems used extensively for protein delivery based on synthetic polymers, liposomes and metal have been replaced as a result of many limitations, e.g. the instability and low loading capacity of liposomes, the disadvantages of low clearance rate and hence enhanced toxicity of metal-based nanocarriers, while those made from synthetic polymers have a limitation of the aggregation of the encapsulated proteins in their inner core [12]. Accordingly, protein NPs and their conjugates have replaced nanocarrier systems, offering the advantages of nanosized structure with good biopharmaceutical characteristics. Also, their production is cost-effective and easy to tailor to meet the specific requirements [13].

The precipitation technique (bottom-up approach) represents the most applicable method for the production of NPs for both small-scale and bulk production, owing to its simplicity, low energy input, low generated temperature and cost-effectiveness compared to the other top-up methods [14]. Zhang et al. prepared a carboxymethyl starch (CMS) microgel system for the control of up taking and releasing proteins (lysozyme) with high pH and high salt-concentration. The microgel particle size was between 25 μm and 45 μm [15]. Xanthan/LYS NPs were produced by precipitation in alkali-coupled thermal condition, and the NPs showed favourable size distribution and stability [16]. On the other hand, when LYS loaded in chitosan (CS) NPs were prepared by the ionic gelation of chitosan and tripolyphosphate (TPP), the CS molecular weight and content, TPP content and initial LYS were reported to have effects on the encapsulation efficiency (EE), release performance and activity of LYS [17]. Moreover, LYS complexed with different concentrations of sodium alginate showed two stages of aggregates with loss of activity based on the alginate content, but antimicrobial activity was recovered upon the addition of calcium chloride [18]. Similarly, LYS was encapsulated in novel cationic polymethacrylate/alginate NPs as a polyelectrolyte carrier system, and the NPs showed high capacity to encapsulate the enzyme, with acceptable polydispersity, biodegradability, high stability and sustained release of LYS. The *in vitro* cytotoxicity of the complex was found to be dose-dependent [19]. Polyelectrolyte multilayers are well-defined nanostructure with some potential applications, such as biomaterial coatings [20]. Also, polyelectrolyte core-shells of bovine serum albumin nanoparticles (BSA-NPs) developed through the layer-by-layer (LBL) technique were used as a carrier system to control the release of ibuprofen. The inner layer was anionically made from poly (sodium-4-styrene) sulphonate, and the cationic outer layer from CS, which enabled interaction with the negatively charged cell membrane and facilitated cell up-take [21].

Accordingly, the arrangement of oppositely charged polyelectrolyte polymers through the LBL approach represents a promising technique for fabrication of micro/nanoparticles and allows their physiochemical and morphological properties to be modified by controlling ionic strength, polymerization degree and the ratio of the polymers [22, 23]. Most importantly, the technique is usually conducted under normal experimental conditions and mainly in an aqueous solution, hence it is suitable to encapsulate proteins and polypeptide drugs [24], and recently the application of three layers of polyelectrolyte polymers in anticancer NPs demonstrated cancer-cell targeting with efficient internalization [25]. In our previous work NPs containing human interferon-α were formulated with the LBL approach by applying chitosan and polystyrene sulphonate [26]. The aim of our present work is to formulate, analyse and optimize core-shell type NPs containing LYS, as well as to write mathematical relationships between process parameters and product parameters.

## Materials and methods

### Materials

Lyophilized lysozyme (MedChemExpress, Hungary), stored frozen (-20°C), was used as a model protein, lyophilized *Micrococcus lysodeikticus* (Sigma-Aldrich, USA) as Gram-positive bacteria to investigate the activity of layered NPs, sodium sulphate (Molar Chemicals Ltd., Hungary) as a precipitating agent, Alginic acid sodium salt (AppliChem GmbH—An ITW Company, Germany) as a layering polymer, sodium hydroxide and hydrochloric acid (Ph. Eur.) as pH adjusters, and all the other reagent s were of analytical grade.

### Methods

#### Preparation of LYS NPs

The preparation of LYS NPs was made according to 2^3^ full factorial design; therefore 8 samples were prepared. Namely, 0.6 g of lyophilized enzyme was dissolved in purified water to obtain 20 g of homogenous aqueous solution, and then each sample was mixed with 4ml of 2M Na_2_SO_4_ solution by using a magnetic stirrer for 1 hour. The 8 samples were centrifuged at 5000 rpm for 15 minutes by using a Hermle Z323K high performance refrigerated centrifuge (Hermle AG, Gossheim, Germany). Upon redispersion the total amount of precipitated LYS aqueous alginate solutions (25 ml) of conc. 0.004 and 0.006%w/v and pH 6 and 10 were added to each sample according to the factorial design. The samples were mixed with a high shear mixer (Ultra-Turrax, Germany) for 15 seconds, followed by mixing for 1 h and 2 h. After the mixing time, the samples were centrifuged again, the supernatant was separated and finally lyophilized at −25°C for 24 h under a pressure of 1.3 Pa, and then kept at 25°C for 24 h for secondary drying to obtain lyophilized powders by using a Scanvac Coolsafe laboratory freeze-dryer (LaboGene, Denmark). The samples were pre-frozen at -10±2°C before lyophilization. The samples were stored at -10±2°C until further investigations.

#### Experimental design

**Fig 1:**
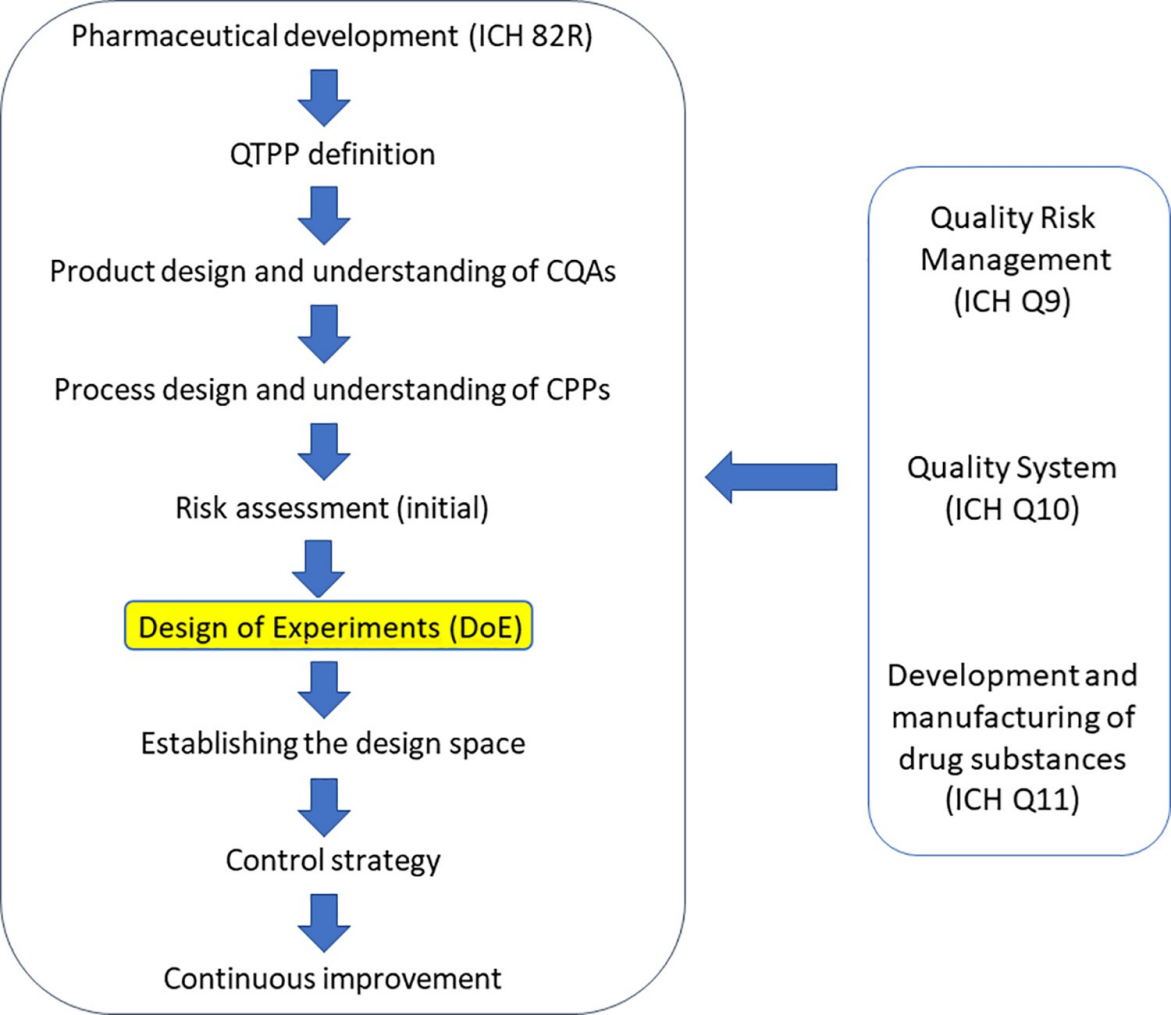
Steps of the extended QbD method.

Following the guidelines of QbD, the steps shown in Fig 1 were performed [27]. Based on the results of the risk analysis, the next step was to select the factors for the factorial experimental design. The factors selected were the concentration of alginate, pH and mixing time, which were used on 2 levels in DOE (Table 1).

**Table 1:** Factor values for samples according to 2^3^ full factorial design.

Sample	c (alg.)%	pH	Mixing time (h)
1	0.004 (-1)	6 (-1)	1 (-1)
2	0.004 (-1)	10 (+1)	1 (-1)
3	0.006 (+1)	6 (-1)	1 (-1)
4	0.006 (+1)	10 (+1)	1 (-1)
5	0.004 (-1)	6 (-1)	2 (+1)
6	0.004 (-1)	10 (+1)	2 (+1)
7	0.006 (+1)	6 (-1)	2 (+1)
8	0.006 (+1)	10 (+1)	2 (+1)

The experiments were conducted according to 2^3^ full factorial design, pH value 6 (-1) and 10 (+1), alginate concentration (0.004% w/v (-1) and 0.006% w/v (+1) and mixing time 1(-1) and 2 (+1) hour) were considered as variable factors. Enzyme activity, particle size, encapsulation efficiency and amount of α-helix were optimization parameters.

The 2^3^ full factorial design (DOE) was applied by Tibco Statistica v13.4.0.14 (Statsoft, Tulsa, OK, USA) software for the evaluation of the effect of factors on the optimization parameters.

#### Precipitation and encapsulation efficiency

The obtained supernatants were carefully removed from the precipitated NPs, then diluted to a suitable range with purified water and the absorption was measured by using a UV spectrometer (ThermoScientific-Genesys 10 S UV-Vis Spectrometer, USA) at lambda max 281 nm. Based on the absorbance, the concentration of unprecipitated NP enzymes was used to calculate precipitation efficiency.

The obtained supernatants were carefully separated from the encapsulated NPs and absorption was measured at 281 nm for each sample based on a pre-recorded calibration line. The concentration of free enzyme NPs was measured for all samples, from which encapsulation efficiency was determined. Encapsulation efficiency (EE%) resulting from the adsorption of alginate on the LYS composite was calculated according to the following equation:

$$EE\%=\frac{\left(total\;amount\;of\;LYS\;(mg)-amount\;of\;LYZ\;in\;the\;supernatant\;sol.(mg)\right)}{total\;amount\;of\;LYS\;(mg)}*100$$ Eq 1

#### Particle size and Zeta potential measurement

The precipitated NPs were adequately diluted, and the particle size of the sample was measured with a Malvern Mastersizer (Malvern Instruments, Malvern, UK). The Zeta potential of the same sample was measured with a Malvern Zetasizer apparatus with three parallel measurements (Malvern Instruments, Malvern, UK).

#### Morphological study

The structure and the morphology of the NPs (after layering) were described with transmission electron microscopy (TEM). The TEM images were made with a FEI TecnaiTM G2 X-Twin HRTEM microscope (FEI Company, Hillsboro, Oregon, US) with accelerating voltage of 200 kV in bright field mode. The samples were suspended in ethanol and dropped onto a carbon film-coated copper grid.

#### Enzyme activity of layered NPs

The activity of the prepared layered nanoparticle samples was recorded by measuring the degradation of lyophilized *Micrococcus lysodeikticus* by using a UV spectrometer (ThermoScientific-Genesys 10 S UV-Vis Spectrometer, USA). 25 mg of lyophilized bacterial cells was dispersed in 100 ml of phosphate buffer (pH 6.8); the basic absorption at 450 nm was around 0.7. The absorptions of the bacterial suspension were measured for 5 minutes before each test to reduce the error arising from bacterial sedimentation. 10 mg of the layered NPs or 10 mg of crude LYS were dissolved in 25 ml of phosphate buffer. 0.1 ml of layered NPs/or crude enzyme solution was added to 2.5 ml of bacterial suspension and shaken for 20 seconds in a quartz cuvette, then the change in bacterial absorption was measured for 5 minutes. LYS activity was calculated from the percentage degradation of the bacterial cells relative to crude LYS activity as a reference.

#### Fourier-transform infrared spectroscopy (FT-IR)

The infrared spectra of the prepared samples and the other excipients were obtained with a FT-IR (Avatar 330 FT-IR ThermoScientific, USA) apparatus, by using the potassium bromide disc method, scanning was run in the wavelength range of 600 to 4000 cm^-1^, the spectra were collected from 64 scans to obtain smooth spectra, at the spectral resolution of 4 cm^-1^ and applying CO_2_ and H_2_O corrections. The SpectraGryph (version 1.2.14.; Dr. Friedrich Menges Software-Entwicklung, Germany) software was used for the second derivation of spectra. For the deconvolution of the second derivatives, the Fityk software was used [28]. After assigning the peaks, the area under the curve was calculated. From these data the α-helix content was determined.

#### Circular Dichroism spectroscopy (CD)

To determine the α-helix content of the initial LYS and the synthesized NPs, circular dichroism (CD) spectra were recorded on an ABL&E-JASCO J-1100 CD spectrometer between 250–190 nm. For the measurements, a 4-opened quartz cuvette with an optical length of 1 cm was used, and the solid samples were dissolved in PBS buffer applying a protein concentration of 0.04 mg/mL. The spectra were corrected with the PBS buffer background. The α-helix content was calculated by using the following equations [29]:

〖MRE〗_208=[Θ]/nand Eq 2

α−helix%=(〖−MRE〗_208−4000)/(33000−4000)*100, Eq 3

where [Θ] is molar ellipticity at ca. 208 nm, n is the number of the amino acids, 33000 is the pure -helix content at 208 nm, while 4000 is the amount of the β-sheet and random coil.

## Results and discussion

### Encapsulation efficiency

After the precipitation step, precipitation efficiency was calculated according to the UV spectra of the supernatant after centrifugation. In this case, average precipitation efficiency was 66.7%, so 0.4002 mg of the precipitated LYS remained in the system. The next step was the layering of alginate with alginate solution of different concentrations and different pH values. These samples were centrifuged again and the supernatant UV-VIS spectra were measured. From these data, the loss of LYS was calculated and summarized with precipitation efficiency, after which encapsulation efficiency can be calculated. EE was between 62.98 and 66.35% in all cases (Table 2). It is a very narrow range because approximately 97% of the entire loss of LYS was lost during the precipitation step. After the layering step, the concentration of LYS of the supernatant was very low after centrifugation. It can be explained by the electrostatic relationship between LYS and polyanionic alginate because the redispersion procedure was performed directly in the alginate solution and LYS could not solve in the buffer because the formation of the alginate layer on the surface of the precipitated LYS started immediately. The alginate layer formed can protect LYS.

**Table 2:** Encapsulation efficiency results.

Sample	Encapsulation efficiency (%)
1	65.17
2	65.76
3	65.65
4	65.87
5	62.98
6	64.21
7	63.64
8	66.35

**Fig 2:**
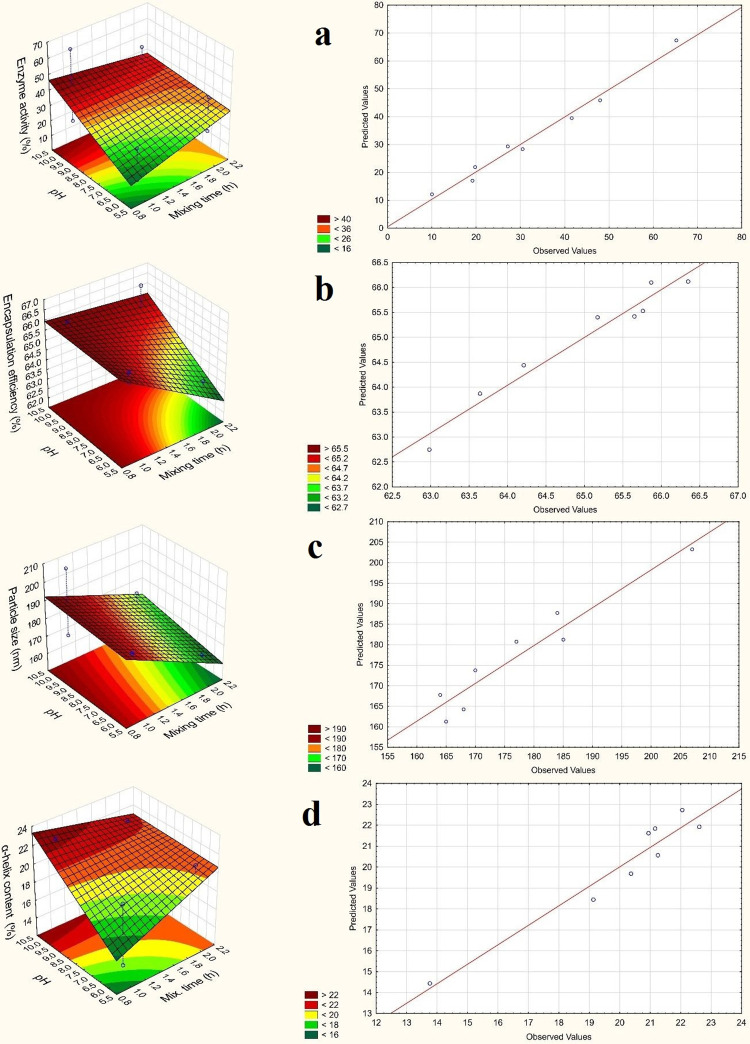
The response surfaces (alginate concentration on zero level) and the predicted values of (a) enzyme activity; (b) EE%; (c) particle size; (d) α-helix content.

The effects of mixing time and pH were important factors, but statistically not significant. There was no great difference between the results because 97% of the loss of LYS was lost after the first centrifugation (first step of formulation) and the first precipitation step was performed with the same method in all cases. Therefore, the values of the coefficients were very low and statistically not significant. An inverse relationship can be seen between mixing time and EE (Fig 2). This can be explained by the starting of the dissolution of LYS from NPs. Therefore, increasing mixing time is not recommended. During a mixing time of 1 h the alginate layer can be formed, which was confirmed by the negative Zeta potential values in all cases. The other important factor is pH, in this case the coefficient was +1.19 (*Eq 4*). Fig 2 reveals that this factor had an effect on EE only in the lower pH range. In the higher pH range dissolution did not start after a mixing time of 2 h. It can be explained with the isoelectric point (IEP) of LYS (pH 11.1) because at around pH 10 near the IEP, the charge difference between LYS and alginate is lower, therefore the degree of the diffusion of LYS is lower in the polyanionic alginate solution. The third factor was alginate concentration, but this effect was very low (0.85). In this case, a low linear relationship was detected between the factor and EE.

The predicted and the observed values can be seen in Fig 2. The predicted values correlate well with the observed values. This mathematical model can be used to show that EE can be predicted well in this range.


$$y=64.95^{*}+0.85x_{1}+1.19x_{2}-1.32x_{3}+0.28x_{1}x_{2}+0.55x_{1}x_{3}+0.78x_{2}x_{3}$$ Eq 4

R^2^ = 0.9867; MS Residual: 0.7875

* statistically significant (p<0.05)

### Results of particle size and Zeta potential

Particle size was measured freshly before lyophilisation with the laser diffraction method. The results were between 164±1 and 207±3 nm in all cases (Table 3). After the precipitation step, the average particle size was 233±3 nm. In each case, it can be seen that the final particle size was smaller than after the first step of preparation. The reason for this is that the polymer layer can result in a more compact NP structure.

**Table 3:** Particle size and zeta potential results.

Sample	Particle size (nm)	Zeta potential (mV)
1	185±1	-18.2
2	170±6	-19.8
3	184±2	-17.6
4	207±3	-18.6
5	164±1	-17.9
6	168±2	-17.5
7	165±1	-18.2
8	177±2	-18.1

It can be seen in Fig 2 that mixing time had the greatest effect on particle size. During mixing, the dissolution of LYS can start from the NPs, and the degradation of the polymer can also start in parallel with this process. This can cause a decrease in particle size. The EE results confirm this because in the case of higher mixing time, EE was lower because of the dissolved LYS during mixing. In this case (x_3_) the coefficient was -9 (*Eq 5*), which means an inverse relationship between particle size and mixing time. The alginate concentration had a smaller effect on particle size. The coefficient was 5.75 (*Eq 5*) and there was a linear relationship between particle size and alginate concentration. It can be explained by the fact that a higher alginate concentration can result in higher layer thickness, which can lead to larger particle size.

The coefficient of pH (x_2_) was 3. The effect of this factor was the lowest, it was not a statistically significant (p<0.005) factor. Fig 2 shows that here the predicted value also correlates well with the observed value, therefore this mathematical model is well applicable to predicting particle size in this range of parameter setting.


$$y=177.5^{*}+5.75x_{1}+3.0x_{2}-9x_{3}+5.75x_{1}x_{2}-3.25x_{1}x_{3}+1.0x_{2}x_{3}$$ Eq 5

R^2^ = 0.9226; MS Residual: 112.5

* statistically significant (p<0.05)

**Fig 3:**
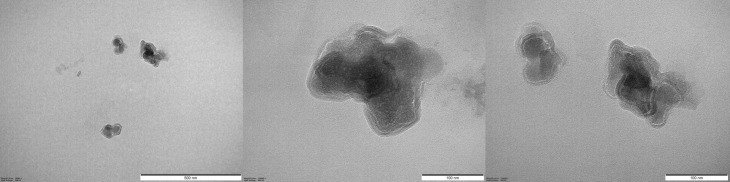
Representative TEM pictures of alginate layered NPs (Sample 2).

The alginate layer on the surface of the precipitated LYS can be observed well (Fig 3). The particle size correlated with the results determined with the Mastersizer based on the TEM, approximately particles around 170 nm are visible. The core-shell structure is clearly visible in the TEM images, which is also supported by the Zeta potential values. The Zeta potential value of the LYS solution was 24±2 mV and decreased to -18.2±0.7 mV for LYS NPs layered with alginate in all cases.

### Enzyme activity

Enzyme activity was measured according to the speed coefficient of the degradation of *Micrococcus lysodeicticus* cell wall. Table 4 shows the enzyme activity results for all samples prepared according to factorial design. Enzyme activity was between 12.1 and 65.2% in each case. The highest value was at pH 10 (+1 level), 0.006% alginate concentration (+1 level) and with a mixing time of 1 h (-1 level).

**Table 4:** The determined enzyme activity (%) values of the different samples.

Sample	Enzyme activity (%)
1	12.10
2	19.18
3	30.49
4	65.20
5	41.60
6	27.14
7	19.77
8	47.99

Based on the statistical evaluation, the effect of factors on enzyme activity can be seen on the response surface. As the response surface of enzyme activity shows, enzyme activity will increase with increasing pH (Fig 2), which can be explained by the IEP of LYS (pH 11.1). If the pH is much lower than the IEP, the secondary structure of the protein may change. The amount of α-helix structure correlates well with enzyme activity. The following equation was obtained as the output of the statistical analysis:

$$y=32.92^{*}+7.94x_{1}+6.96x_{2}+1.20x_{3}+8.78x_{1}x_{2}-8.19x_{1}x_{3}-3.51x_{2}x_{3}$$ Eq 6

R^2^ = 0.9838; MS Residual: 28.69

* statistically significant (p<0.05)

In this case, only b_0_ was a statistically significant factor, which means the average value. Alginate concentration (x_1_) had the largest effect on enzyme activity (7.94), and pH (x_2_) also had a great effect (6.96) (*Eq 6*). In this range mixing time had no significant effect. The two-way interaction coefficients were also high for x_1_x_2_ and x_1_x_3._ The correlation between the predicted and the observed values can be seen in Fig 2. It can show the accuracy of the calculated mathematical model for enzyme activity. This means that enzyme activity can predict well in this range with the application of this mathematical model.

### FTIR and secondary structure analysis

**Fig 4:**
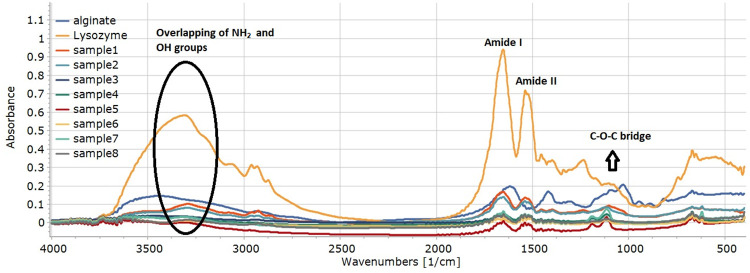
FT-IR spectra of the different samples.

The samples were analysed with FTIR in KBr pastilles. The amide I, II and III characteristic peaks of proteins can be well assigned in each case (Fig 4). The amide I region can be found between 1700–1615 cm^-1^ [30].

**Fig 5:**
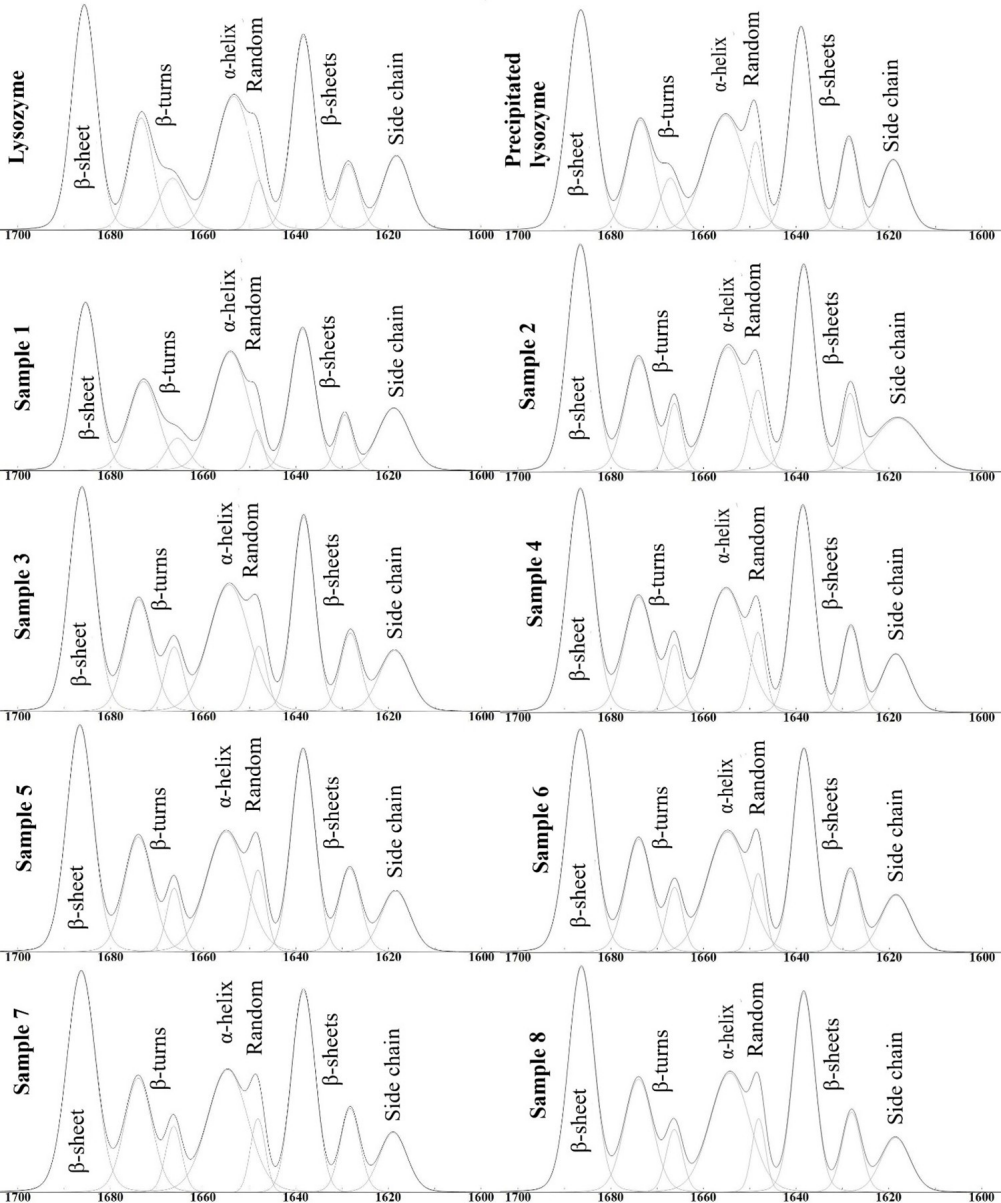
Deconvolution of FT-IR spectrum of LYS, precipitated LYS and the samples.

After the second derivation of the 1700–1600 cm^-1^ region, the deconvolution of the peaks was performed, the results of which are shown in Fig 5. Seven main peaks were found in this region. At 1685 cm^-1^, 1637 cm^-1^ and 1629 cm^-1^ the β-sheets, at 1672 cm^-1^ and 1666 cm^-1^ the β-turns, at 1654 cm^-1^ the α-helix right next to 1648 cm^-1^ as random, at 1618 cm^-1^ the side chain structure was specific.

The amount of α-helix or other structures can be calculated from the area of the peaks. In Table 4 the amount of α-helix can be seen. For the raw material LYS, the α-helix content was 22.69%, which is lower than the literature data (40% [31]; 34% in phosphate buffer pH 5.1 [32]; 40% in D_2_O solution [33]; 30% in water [34]). This may be due to freeze-dried LYS because this product may be more sensitive to environmental parameters than spray-dried LYS. The α-helix content of precipitated LYS was 19.66% (Table 5). The α-helix content of the samples was higher than this value in all cases except for Sample 1 and Sample 5. In these cases, both alginate concentration and pH were at minimum levels. The reason for this may be that at pH 6 (-1 level) the alginate concentration (-1 level) is too low to stabilize the NPs, but if mixing time increases to 2 h, the α-helix content is also higher (Sample 5). In all cases, if the pH was 6, the α-helix was lower than at pH 10. This can be explained with the IEP of LYS (11.1) because the α-helix content near the IEP can be higher than at lower pH. The effect of pH and mixing time as well as the tendency of the α-helix content can also be observed on the response surface (Fig 2). However, Szigeti et al. concluded that pH changes in the 6.0–8.7 range cannot influence the secondary structure of the protein significantly, but in this study only the horseradish peroxidase was investigated [35].

**Table 5:** The α-helix content of the samples.

Sample	Content of α-helix (%)
LYS	22.69
Precipitated LYS	19.66
1	13.76
2	22.61
3	20.37
4	22.05
5	19.13
6	21.16
7	20.94
8	21.25

This tendency correlates very well with the enzyme activity results (Fig 2). It can be seen that enzyme activity increases with the α-helix content. In the course of the statistical evaluation, there was no statistically significant (p<0.05) factor. The effects of all factors were positive (*Eq 7*), which means a linear relationship between the factors and the optimization parameter. The coefficient of pH was the highest value (+1.61), which can be explained by the fact that the secondary structure of proteins may change with changing pH. We found that the amount of α-helix increases slightly with increasing alginate concentration and mixing time.


$$y=20.16^{*}+0.99x_{1}+1.61x_{2}+0.46x_{3}-1.11x_{1}x_{2}-0.52x_{1}x_{3}+1.02x_{2}x_{3}$$ Eq 7

R^2^ = 0.9318; MS Residual: 3.71

* statistically significant (p<0.05)

### CD spectroscopy

**Fig 6:**
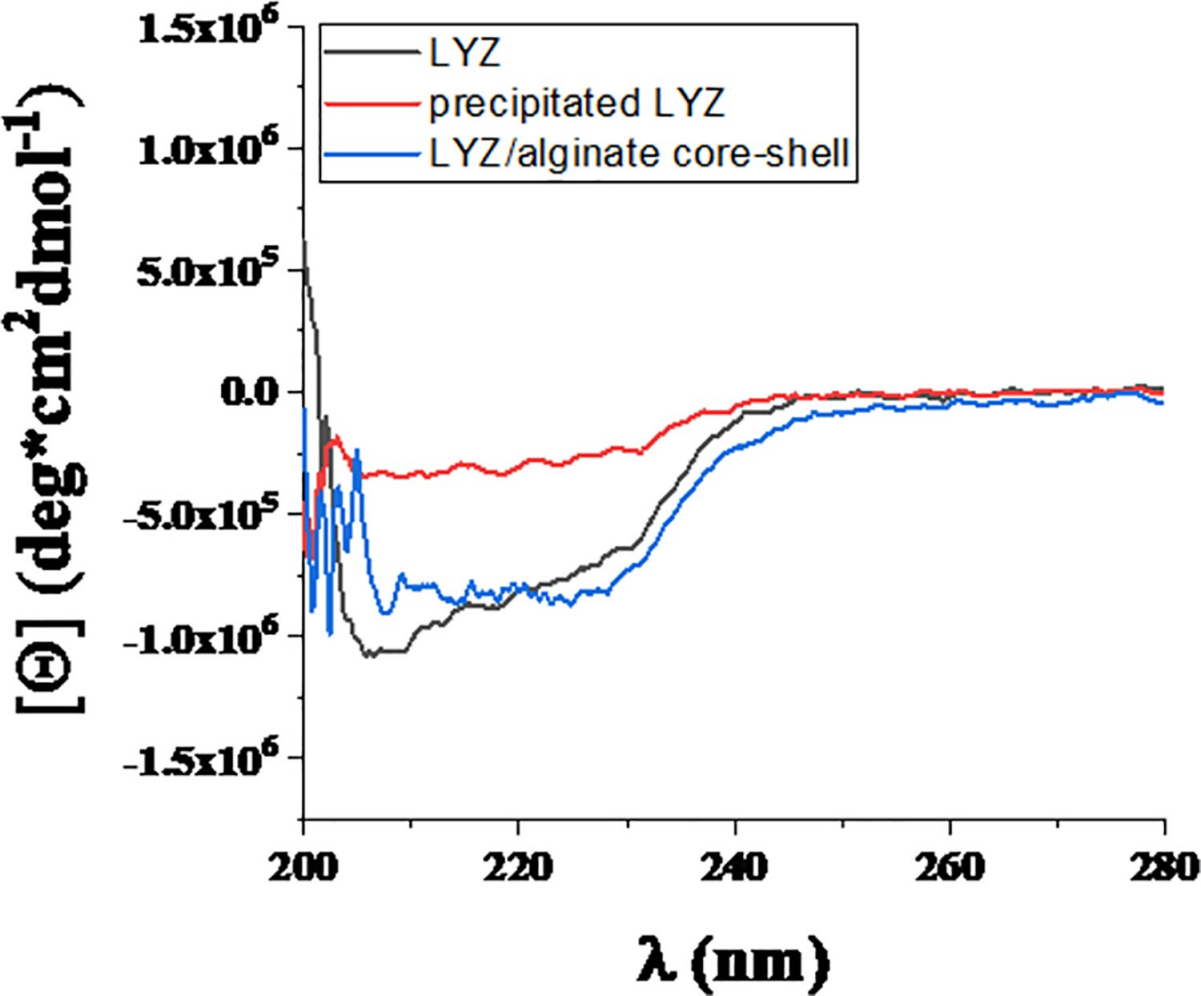
Results of CD spectroscopy for LYS, precipitated LYS and core-shell NPs.

As shown in Fig 6, the spectra of LYS NPs and alginate-LYS core-shell nanostructures consist of more disordered secondary structures than the initial LYS. Namely, the α-helix content is 41.79%, 22.75% and 35.12% for LYS, precipitated LYS and core-shell NPs, respectively. Based on the CD measurements, the protein chain unfolds during the synthesis of LYS-based NPs, while the alginate shell causes a more compact structure because it wraps and compresses the chains of the precipitated protein.

Comparing the results obtained from the FTIR spectra, it can be seen that there is only a small difference between the precipitated LYS and a major difference between the α-helix results for the starting LYS (22.69%) and NPs (21.16%). The reason for this may be that the FTIR measurement was performed in solid state of protein, while CD spectroscopy was measured in liquid. Therefore, only the precipitated LYS had a similar value for the α-helix contents (3% difference) because in this case the precipitated LYS was also present as solid particles in the liquid during the CD measurements.

## Conclusions

In this study, a simple procedure and analysis for the preparation of core-shell NPs containing LYS were presented. The secondary structure of all samples was determined and statistically evaluated. As regards enzyme activity and the content of α-helix, pH was the most important factor because the α-helix secondary structure is present to a greater extent close to that of IEP of LYS. These optimization parameters correlate well each other. During the formulation of NPs containing LYS pH 10 is recommended. The coefficient of the effect of mixing time was the highest for encapsulation efficiency and particle size, since the dissolution of LYS started during mixing, therefore a mixing time of 1 h is recommended during formulation. The results of the α-helix content of FTIR and CD measurements were very similar for the precipitated LYS due to the solid state of LYS. In the case of alginate layered and raw material LYS, the difference was very high because of the liquid form during the CD measurements. Mathematical models were set up successfully in accordance with the QbD guidelines, which can be used to predict future optimization parameters and design space determination in this range. In summary, this information may help the design of the formulation in the future because it was a very simple composition with a minimal number of excipients applied, therefore only the factors can affect the optimization parameters no other effects should be considered.

## Supporting information

S1 File (PDF)
