# Supplementary material for: Optimization of layering technique and secondary structure analysis during the formulation of nanoparticles containing lysozyme by quality by design approach
Source: PLoS One. 2021 Dec 9;16(12):e0260603. doi: 10.1371/journal.pone.0260603 (PMC8659335; doi:10.1371/journal.pone.0260603)
Supplement: S1 File — (PDF) [file pone.0260603.s001.pdf]

S1 Table Statistical results of encapsulation efficiency

Factor	Effect Estimates; Var.:Encapsulation efficiency (%); R-sqr=.95783; Adj:.70482 (Spreadsheet19) 2**(3-0) design; MS Residual=.4278125 DV: Encapsulation efficiency (%)									
	Effect	Std.Err.	t(1)	p	-95.% Cnf.Limt	+95.% Cnf.Limt	Coeff.	Std.Err. Coeff.	-95.% Cnf.Limt	+95.% Cnf.Limt
Mean/Interc.	64.95375	0.231250	280.8811	0.002267	62.01544	67.89206	64.95375	0.231250	62.01544	67.89206
(1)c alg (%)	0.84750	0.462500	1.8324	0.318026	-5.02912	6.72412	0.42375	0.231250	-2.51456	3.36206
(2)pH	1.18750	0.462500	2.5676	0.236440	-4.68912	7.06412	0.59375	0.231250	-2.34456	3.53206
(3)Mix. time (h)	-1.31750	0.462500	-2.8486	0.214926	-7.19412	4.55912	-0.65875	0.231250	-3.59706	2.27956
1 by 2	0.27750	0.462500	0.6000	0.655958	-5.59912	6.15412	0.13875	0.231250	-2.79956	3.07706
1 by 3	0.55250	0.462500	1.1946	0.443698	-5.32412	6.42912	0.27625	0.231250	-2.66206	3.21456
2 by 3	0.78250	0.462500	1.6919	0.339838	-5.09412	6.65912	0.39125	0.231250	-2.54706	3.32956

S2 Table Statistical results of particle size

Factor	Effect Estimates; Var.:Particle size (nm); R-sqr=,92263; Adj:,45839 (Spreadsheet19) 2**(3-0) design; MS Residual=112,5 DV: Particle size (nm)									
	Effect	Std.Err.	t(1)	p	-95,% Cnf.Limt	+95,% Cnf.Limt	Coeff.	Std.Err. Coeff.	-95,% Cnf.Limt	+95,% Cnf.Limt
Mean/Interc.	177.5000	3.750000	47.33333	0.013448	129.852	225.1483	177.5000	3.750000	129.8517	225.1483
(1)c, alg. (%)	11.5000	7.500000	1.53333	0.367904	-83.797	106.7965	5.7500	3.750000	-41.8983	53.3983
(2)pH	6.0000	7.500000	0.80000	0.570447	-89.297	101.2965	3.0000	3.750000	-44.6483	50.6483
(3)Mix. time (h)	-18.0000	7.500000	-2.40000	0.251332	-113.297	77.2965	-9.0000	3.750000	-56.6483	38.6483
1 by 2	11.5000	7.500000	1.53333	0.367904	-83.797	106.7965	5.7500	3.750000	-41.8983	53.3983
1 by 3	-6.5000	7.500000	-0.86667	0.545396	-101.797	88.7965	-3.2500	3.750000	-50.8983	44.3983
2 by 3	2.0000	7.500000	0.26667	0.834095	-93.297	97.2965	1.0000	3.750000	-46.6483	48.6483

S3 Table Statistical results of enzyme activity

Factor	Effect Estimates; Var.:Enzyme activity (%); R-sqr=,98686; Adj:,908 (Spreadsheet19) 2**(3-0) design; MS Residual=28,69031 DV: Enzyme activity (%)									
	Effect	Std.Err.	t(1)	p	-95,% Cnf.Limt	+95,% Cnf.Limt	Coeff.	Std.Err. Coeff.	-95,% Cnf.Limt	+95,% Cnf.Limt
Mean/Interc.	32.9213	1.893750	17.38416	0.036580	8.8589	56.98363	32.92125	1.893750	8.8589	56.98363
(1)c, alg. (%)	15.8775	3.787500	4.19208	0.149077	-32.2473	64.00225	7.93875	1.893750	-16.1236	32.00113
(2)pH	13.9125	3.787500	3.67327	0.169211	-34.2123	62.03725	6.95625	1.893750	-17.1061	31.01863
(3)Mix. time (h)	2.4025	3.787500	0.63432	0.640134	-45.7223	50.52725	1.20125	1.893750	-22.8611	25.26363
1 by 2	17.5575	3.787500	4.63564	0.135259	-30.5673	65.68225	8.77875	1.893750	-15.2836	32.84113
1 by 3	-16.3725	3.787500	-4.32277	0.144725	-64.4973	31.75225	-8.18625	1.893750	-32.2486	15.87613
2 by 3	-7.0275	3.787500	-1.85545	0.314696	-55.1523	41.09725	-3.51375	1.893750	-27.5761	20.54863

S4 Table Statistical results of α-helix content

Factor	Effect Estimates; Var.:α-helix content (%); R-sqr=.9318; Adj:.52257 (Spreadsheet19) 2**(3-0) design; MS Residual=3.712812 DV: α-helix content (%)									
	Effect	Std.Err.	t(1)	p	-95.% Cnf.Limt	+95.% Cnf.Limt	Coeff.	Std.Err. Coeff.	-95.% Cnf.Limt	+95.% Cnf.Limt
Mean/Inter c.	20.15875	0.681250	29.59083	0.021506	11.5026	28.81485	20.15875	0.681250	11.50265	28.81485
(1)c alg (%)	1.98750	1.362500	1.45872	0.382578	-15.3247	19.29970	0.99375	0.681250	-7.66235	9.64985
(2)pH	3.21750	1.362500	2.36147	0.255011	-14.0947	20.52970	1.60875	0.681250	-7.04735	10.26485
(3)Mix. time (h)	0.92250	1.362500	0.67706	0.621105	-16.3897	18.23470	0.46125	0.681250	-8.19485	9.11735
1 by 2	-2.22250	1.362500	-1.63119	0.350115	-19.5347	15.08970	-1.11125	0.681250	-9.76735	7.54485
1 by 3	-1.03750	1.362500	-0.76147	0.585688	-18.3497	16.27470	-0.51875	0.681250	-9.17485	8.13735
2 by 3	-2.04750	1.362500	-1.50275	0.373796	-19.3597	15.26470	-1.02375	0.681250	-9.67985	7.63235
